# Incidence of Bacteriobilia and the Correlation with Antibioticoprophylaxis in Low-Risk Patients Submitted to Elective Videolaparoscopic Cholecystectomy: A Randomized Clinical Trial

**DOI:** 10.3390/antibiotics12101480

**Published:** 2023-09-25

**Authors:** Marcos Alberto Pagani, Pedro Meira Dolfini, Beatriz Flávia de Moraes Trazzi, Maria Ines Meira Dolfini, William Saranholi da Silva, Eduardo Federighi Baisi Chagas, Carlos Henrique Bertoni Reis, João Paulo Galletti Pilon, Bruna Trazzi Pagani, Rodrigo Tavore Strasser, Claudemir Gregório Mendes, Fausto Tucunduva Vernaschi, Daniela Vieira Buchaim, Rogerio Leone Buchaim

**Affiliations:** 1 UNIMAR Beneficent Hospital (HBU), Medical School, University of Marilia (UNIMAR), Marilia 17525-160, Brazil; 2 Department of General Surgery, Regional Hospital, University of West Paulista (UNOESTE), Presidente Prudente 19050-680, Brazil; 3 Dentistry School, University of Marilia (UNIMAR), Marilia 17525-902, Brazil; 4 Morphofunctional Department, University of West Paulista (UNOESTE), Presidente Prudente 19050-920, Brazil; 5 Postgraduate Program in Structural and Functional Interactions in Rehabilitation, University of Marilia (UNIMAR), Marilia 17525-902, Brazil; 6 Interdisciplinary Center on Diabetes (CENID), University of Marilia (UNIMAR), Marilia 17525-902, Brazil; 7 Postgraduate Program in Speech Therapy, Sao Paulo State University (UNESP—Universidade Estadual Paulista), Marilia 17525-900, Brazil; 8 Faculty of Pharmacy and Biomedicine, University of Marilia (UNIMAR), Marilia 17525-902, Brazil; 9 Medical School, Educational Foundation of the Municipality of Assis (FEMA), Assis 19807-130, Brazil; 10 Medical School, University Center of Adamantina (UNIFAI), Adamantina 17800-000, Brazil; 11 Graduate Program in Anatomy of Domestic and Wild Animals, Faculty of Veterinary Medicine and Animal Science, University of Sao Paulo, Sao Paulo 05508-270, Brazil; 12 Department of Biological Sciences, Bauru School of Dentistry (FOB/USP), University of Sao Paulo, Bauru 17012-901, Brazil

**Keywords:** antibiotic prophylaxis, bile, cholelithiasis, cholecystectomy, video-assisted surgery, randomized clinical trial

## Abstract

Cholelithiasis has a major impact on global health and affects an average of 20% of the Western population. The main risk factors are females, age over 40 years, obesity and pregnancy. Most of the time it is asymptomatic, but when there are symptoms, they are generally nonspecific. Bile was considered sterile, but today it is known that it contains a complex bacterial flora, which causes biofilm in the gallbladder and gallstones. Among the main bacteria associated with cholelithiasis are *Pseudomonas aeruginosa*, *Escherichia coli*, *Klebsiella pneumoniae*, species of *Enterococcus* spp. and *Acinetobacter* spp. Antibiotic prophylaxis is used in an attempt to reduce postoperative infections, especially at the surgical site. However, some authors found no relationship between the use of antibiotic prophylaxis and a lower risk of surgical site infection. Thus, the aim of this double-blind randomized clinical trial was to compare the existence or not of bacteriobilia in patients at low anesthetic risk who underwent videolaparoscopic cholecystectomy, and its correlation with the use of prophylactic antibiotics. This study included 40 patients between 18 and 65 years old, diagnosed with cholelithiasis, symptomatic or not, with low anesthetic risk classified by the American Society of Anesthesiology in ASA I or ASA II, without complications or previous manipulation of the bile duct, who underwent elective video cholecystectomy, divided into two groups: Experimental Group A (*n* = 20), which received 2 g of Cephalotin (first-generation Cephalosporin, Keflin^®^, ABL antibiotics, Cosmópolis, Brazil) during anesthetic induction, and Control Group B (*n* = 20), where no antibiotics were administered until bile collection. After the procedure, a bile sample was collected and culture and antibiogram were performed. In the sample, 22 (55%) were classified as ASA I and 18 (45%) as ASA II. It was observed that 81.8% of the patients who had a positive culture did not use antibiotics, against 18.2% of those who used prophylaxis. When comparing patients regarding anesthetic risk, ASA I patients had a positive culture in 9.1% of cases, against 90.9% in patients classified as ASA II. It was concluded that patients with higher anesthetic risk (ASA II) have a higher chance of bacteriobilia and benefit from antibiotic prophylaxis when compared to patients with lower anesthetic risk (ASA I).

## 1. Introduction

Cholelithiasis is a disease with a high incidence in the Western population, affecting 10 to 20% of adults, causing a great impact on health systems [1,2,3]. It presents as the main risk factor for female individuals, aged over 40 years, with obesity, in addition to genetic, environmental and metabolic conditions. Although most patients are asymptomatic, among the main symptoms reported is abdominal pain with sudden onset located in the upper right quadrant of the abdomen, with or without nausea and vomiting, and its main complications include infection, gangrene and perforation [4,5,6,7,8,9].

In the United States, more than 700,000 cholecystectomies are performed, and every year more patients are operated on in an attempt to avoid complications from cholelithiasis. In Brazil, most procedures are still performed by laparotomy (88%) due to inequality in the distribution of devices and services. Laparoscopic cholecystectomy has several advantages over open surgery, such as a smaller incision, shorter hospital stay, faster return to daily activities, and a lower risk of wound infection [10,11,12].

The healthy biliary tree was considered sterile, but new studies indicate that the gallbladder contains a very complex bacterial flora, with several possible colonization routes. Culture results demonstrate the existence of biofilm-forming bacteria in the gallbladder, bile, and gallstones, including *Pseudomonas aeruginosa*, *Escherichia coli* (*E. coli*), *Klebsiella pneumoniae*, *Enterococcus* spp. and *Acinetobacter* spp. [13,14].

Although controversial, antibiotic prophylaxis in patients undergoing video cholecystectomy is indicated by some authors in order to reduce the risk of surgical site infection [15,16]. However, recent studies have not found a significant difference in patients with prophylactic antibiotic therapy or the use of a placebo in preventing surgical site infection in patients electing for video cholecystectomy [17,18,19].

This study is justified by the fact that, if it is possible to avoid antibiotic prophylaxis, a reduction in costs can be achieved, in addition to reducing the possibility of bacterial resistance to antibiotics. Therefore, to meet the objectives of the study, a double-blind randomized clinical trial was carried out to compare the existence or not of bacteriobilia in patients at low anesthetic risk, submitted to videolaparoscopic cholecystectomy, and its correlation with the use of prophylactic antibiotics.

## 2. Results

Forty participants were included in the study, 34 female (85%) and 6 male (15%). The postoperative period of all patients in the study was followed up for 60 days. No patient had a relevant change for the description or intercurrence that required intervention. The mean age of the sample was 44.9 ± 11.3 years, with a minimum of 23 and a maximum of 63 years. No significant differences (*p* value = 0.127) were observed between participants with positive culture (49.3 ± 10.6 years) and negative culture (43.2 ± 11.3 years). Furthermore, in the sample, the participants were classified by the anesthesia service as ASA I in a total of 22 (55%) of the patients, and as ASA II in a total of 18 (45%) of the patients, divided between the groups that are statistically similar (Table 1).

Among the evaluated patients, 2 of them were affected by obesity (27.5%), 9 with hypothyroidism (22.5%), 7 with diabetes (7%), 8 smokers (20%) and 11 with hypertension (27.5%). Eleven patients had bacterial growth in bile cultures (27.5%), regardless of group. No significant differences were observed in the frequency distribution of sex, ASA, hypertension, diabetes, hypothyroidism, smoking and obesity, which indicates that the groups are similar. However, the experimental group (A) showed a significantly lower proportion of positive culture than the control group (B) (Table 1).

Among the patients who had a positive culture, 18.2% received antibiotic prophylaxis and 81.8% did not receive prophylaxis. Among patients classified as ASA I, 9.1% had a positive culture, and of patients classified as ASA II, 90.9% had a positive culture. Classification according to the ASA presented a fourteen times greater chance (14×) of a positive culture in ASA II patients (Odds Ratio, Odds > 1) compared to ASA I, as shown in Table 2.

In Table 3, the regression analysis confirms that both ASA and the use of prophylactic antibiotics significantly modified the possibility of a positive culture. In ASA II patients, there was an increase in the probability of having a positive culture. However, the use of antibiotics is a protective factor and reduces the chance of a positive culture. Together, the ASA and the antibiotic explain 42.6% (R^2^) of the variance in the probability of having a positive culture.

A positive biliary culture was observed in a total of 11 patients (27.5%). Group A, with a total number of 20 patients, who received antibiotic prophylaxis, presented 2 positive samples for bacteriobilia, which represents 10% of the analyzed samples, while Group B, also with 20 patients, who did not receive any antibiotics, presented positive samples for bacteriobilia in 45% of the analyzed cases. This relationship showed statistical significance (*p* < 0.05)—(*p* = 0.0035).

Nineteen patients were 45 years old or older; of these, 7 (36.8%) had a positive biliary culture, while 21 patients were younger than 45 years old, and, of these, 4 (19%) had a positive biliary culture (*p* = 0.8982). Of the 6 male patients, only 1 (16.6%) had a positive biliary culture, while among the 34 female patients evaluated, 10 (29.4%) had a positive biliary culture (*p* = 0.2281). None of these relationships showed statistical significance.

In the patients who did not undergo prophylactic antibiotics (Group B), 9 patients had positive cultures with different microorganisms, 3 of which were *Coagulase-negative Staphylococcus*, 2 for *Staphylococcus aureus*, 2 for *Klebsiella pneumoniae*, 1 for *Enterococcus faecalis* and 1 for alpha-hemolytic *Streptococcus*, in addition to one patient who had a mixed culture with *Candida albicans*.

In these cultures, the antibiogram showed bacteria sensitive to all antibiotics tested. In the group submitted to antibiotic prophylaxis (Group A), only 2 patients had a positive culture, with *Proteus vulgaris* + *Escherichia coli* in the same culture and alpha-hemolytic *Streptococcus* in another, and the antibiogram showed resistance to the antibiotic used in this study, Cephalin (1st generation Cephalosporin).

Table 4 shows the association analysis in the rate of complications with ASA, use of antibiotics (group) and culture. No significant association was observed between the complication rate and ASA, use of antibiotics (experimental) and culture, although the dataset showed a higher proportion of ASA II and control groups among patients with severe complications. However, among uncomplicated patients, the ASA and group distribution were very similar.

## 3. Discussion

We thought of carrying out this study to evaluate the correlation between the use of antibiotics before video cholecystectomy surgery and the presence of bacteriobilia. It was observed that prophylactic antibiotic therapy becomes important in patients with higher surgical risk. Cholelithiasis is a very common pathology in the population and, in most cases, it does not show symptoms, but when they do appear, they can be of different types. Laparoscopy remains the gold standard for the surgical management of symptomatic or complicated patients [6,20,21].

In this study, in all research participants, we chose to collect bile in a sterile surgical environment, immediately after removal of the gallbladder (extracorporeal). Removal inside the abdominal cavity could lead to extravasation of the contents inside the abdominal cavity, increasing surgical time and risk, due to the need to clean the cavity. In addition, there would be a risk of gallstones remaining in the cavity, which could lead to the formation of granulomas [22].

During the experiment, we found a predominance of female patients diagnosed with cholelithiasis (85%), with a mean age of 44.9 ± 11.3, which is in line with the main risk factors for the pathology; however, few patients were seen with obesity (5%), going against the same factors [1,5,8,23].

Similar recent studies have demonstrated the association of a positive bile culture with advanced patient age, suggesting that these patients should be prescribed a prophylactic antibiotic preoperatively [24,25]. In this study, we found no difference in age (*p* value = 0.127) between patients with positive and negative cultures. Bacteriobilia has been associated with the presence of gallstones, and the bacteria isolated from the collected bile cultures are similar to the intestinal flora. Studies have shown a higher incidence of bacteriobilia in older patients, and our clinical research has shown a higher prevalence in patients with ASA II compared to ASA I patients, suggesting the need for antibiotic prophylaxis in this group of patients [26,27].

**Figure 1:**
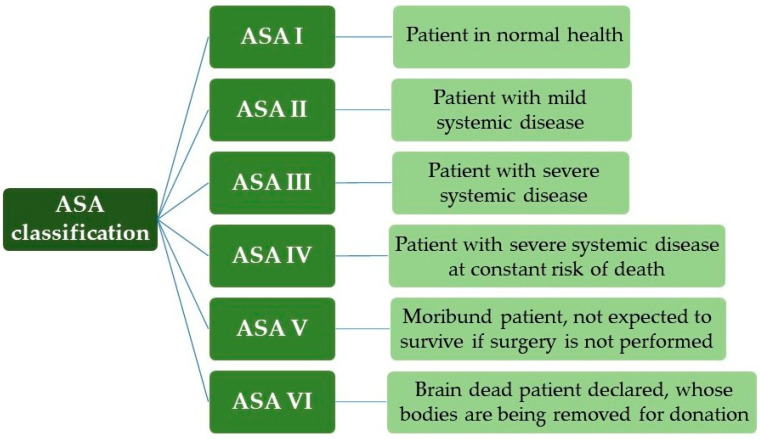
ASA classification (American Society of Anesthesiologists).

For the surgical risk classification, the anesthetic risk categories and the physical status classification according to the American Society of Anesthesiologists (ASA) were used, an important reference for the pre-anesthetic evaluation of the patient [28,29]. Thus, it is used in several studies due to its close relationship with anesthetic morbidity and mortality, as shown in Figure 1.

With a patient classified as ASA I, we can exemplify the healthy individual, non-smoker, with no or minimal use of alcohol. ASA II are patients without significant functional limitations, that is, smokers, pregnancy, obesity (30 < body mass index, BMI < 40), controlled systemic arterial hypertension (SAH) and diabetes mellitus (DM). With ASA III, the patient has significant functional limitations, uncontrolled SAH and DM, morbid obesity, Chronic Obstructive Pulmonary Disease (COPD), moderate heart failure and previous acute myocardial infarction (AMI) (>3 months). ASA IV occurred in recent AMI (<3 months), sepsis, severe heart failure and previous cerebral ischemia, and, for ASA V, ruptured aneurysm, massive trauma and multiple organ dysfunction [30,31]. 

In this study, patients classified by anesthetic risk ASA I and II were selected because, in the gastroenterology sector where participants were recruited (UNIMAR Beneficent Hospital—HBU, Medical School, University of Marilia, Brazil), the number of patients with ASA classification III and IV is low. In addition, the surgical risk of these patients is greater, leading to a possible need for beds in Intensive Care Units (ICU) and longer hospital stays. It should also be considered that in March 2020, with the COVID-19 pandemic, all elective surgeries were canceled.

Previous studies associated a greater chance of bacteriobilia in patients with higher anesthetic risk (ASA II), which was also found in this study, with 90.9% of patients with positive culture, in addition to demonstrating that the prophylactic antibiotic reduces the chance of positive culture in these patients [1,26].

It was observed that the association of ASA II and non-use of antibiotics increased the risk of a positive culture, and that ASA II patients who underwent antibiotic prophylaxis had a lower chance of a positive culture. However, it cannot be affirmed that the use of prophylactic antibiotics immediately before the incision is responsible for bacterial elimination from the bile, which can also presume that the positive bile culture is a coincidence. Prophylaxis with antibiotics can reduce the risk of bacterial dissemination and, consequently, of post-surgical infections. In similar studies, it was suggested that the use of prophylactic antibiotics in elderly people with comorbidities would be indicated to reduce the risk of infection [32,33].

The evaluation of the association of comorbidities, alone, with a positive culture did not show relevance with any of the evaluated comorbidities, which contradicts data from similar studies, which showed an increased risk of positive culture in patients with diabetes mellitus, for example [26,34].

In this study, the positive biliary culture was 27.5%, a result close to that found by Yaqin et al. (1978), which was 25.7% [35], and Al Harbi et al. (2001), who obtained a positive biliary culture in 25% of the cases [36]. When comparing the groups (Groups A and B), there was an increase in positive bile culture in the group that did not receive prophylactic antibiotics (Group B). Group A, which received prophylactic antibiotics, had a rate of 10% of bacteriobilia, while group B, which did not receive antibiotics, had a higher rate of 45% of bacteriobilia.

*Coagulase-negative Staphylococcus* was considered the most common organism in this study found in 23.08% of the positive samples, followed by *Staphylococcus aureus*, *Klebsiella pneumoniae* and alpha-hemolytic *Streptococcus* with 15.38%. *Escherichia coli*, *Proteus vulgaris*, *Enterococcus faecalis* and *Candida albicans* appeared in the samples at an incidence of 7.69%. These results differ from results found in other studies [36,37,38] where the predominant incidence in the three studies is of *Escherichia coli* ranging from 28.1% to 45.07%, followed by *Klebsiella pneumoniae*.

In the antibiogram, the bacteria found were sensitive to first, second and third-generation cephalosporins (Cephalotin, cefuroxime and cefitriaxone, respectively), except for the strain of *Proteus vulgaris* which was resistant to first and second-generation cephalosporins. Regarding quinolones, all strains were sensitive to ciprofloxacin and only one strain of *Streptococcus* spp. was resistant to levofloxacin. The highest index of resistance found was for ampicillin with 38.4% of resistant samples. All evaluated strains were also sensitive to clindamycin, clarithromycin, oxacillin, linezolid and vancomycin. This pattern found in multisensitive strains follows other studies [1,36,39,40].

Thus, in view of what is exposed in this manuscript, previous studies corroborate our results. In a systematic review and meta-analysis, Yang et al. (2021) [41] aimed to investigate whether the perioperative use of prophylactic antibiotics during elective laparoscopic video cholecystectomy could reduce the incidence of postoperative infection. For this, they analyzed 14 randomized clinical trials involving 4360 patients. The results indicated that in low-risk patients undergoing cholecystectomy, prophylactic antibiotics reduce the incidence of surgical site infections. In a retrospective study, Ely et al. (2020) [42] reviewed elective laparoscopic cholecystectomies from the American College of Surgeons National Surgical Quality Improvement Program database for the period of 2016–2017. They observed that respiratory infection occurred in 1.0% of cases and the incidence of surgical infection (when elective) is very low, and identified several characteristics of patients strongly restricted to respiratory infection such as diabetes, hypertension, smoking, ASA class > 2, operative time (for minute) and class 4 wound.

According to the data in Table 4, which analyzed the association of absolute (*n*) and relative (%) frequency distribution of the rate of complications with positive culture, use of antibiotics and ASA, there were more severe complications in the postoperative period of the ASA II patients (*n* = 4; 80%) and of the Control Group (Group B—without antibiotics, *n* = 4; 80%). Due to the data observed in Table 4, it can be emphasized that the complication rates did not differ significantly between the comparator group (control B) and the experimental group A. Therefore, our results are consistent with studies that demonstrate that antibiotics reduce postoperative complications in cholecystectomy surgeries (removal of the gallbladder) [7,43,44].

A limitation of this study can be considered the use of a single class of antibiotic, with no comparison of efficacy with other drugs. This fact raises questions for future research with the inclusion of a larger number of patients and testing of other antibiotics, comparing their effectiveness in reducing or excluding bacteria in the bile, in the search to find a more specific antibiotic for each group, individualizing the treatment. In addition, we can consider the limitation that the intent of the study design was to compare the existence of bacteriobilia and its correlation with prophylactic antibiotics. The presence or absence of bacteria does not demonstrate causation of complications such as surgical site infections.

## 4. Materials and Methods

A double-blind randomized clinical trial was performed. Blinding occurred for patients, who were not aware of which group they were included. Blinding also occurred for the team of technicians in the analysis laboratory. To ensure groups of the same size, randomization occurred by changing the entry into groups, with the first patient being defined by drawing lots.

The sample size was estimated to analyze the association between the presence of bacteriobiliary and the use of prophylactic antibiotics. The minimum sample size is 32 sample elements for a type I error margin (α) of 5%, a study power of 80% and a degree of freedom and a large effect size (0.50). Considering a possible sample loss, 48 sample elements were selected in the study [35]. Sample size calculations were performed using the G*Power software, version 3.1.9.2 (Franz Faul, Universität Kiel, Kiel, Germany).

The effect size observed in the study for the results of the association analysis between the presence of a positive culture and the use of antibiotics was 0.57. Therefore, the value is higher than the effect size predicted in the sample size calculation, suggesting that the estimated sample size was adequate. Considering the size of the observed effect, there was a power of 89% for analyzing the relationship between culture and antibiotic use (Table 5).

This single-centre study was carried out at UNIMAR Beneficent Hospital—HBU, Medical School, University of Marilia (Brazil), in partnership with the Interdisciplinary Master’s Program in Structural and Functional Interactions in Rehabilitation (UNIMAR), with the participation of professionals from different areas of health and residents of Surgery General and Gastroenterology.

This study was carried out with 48 patients diagnosed with gallstones, from October 2019 to February 2020 (longitudinal observational). The research protocol was approved by the Research Ethics Committee of the University of Marília (UNIMAR) under number 3,545,220 on 30 August 2019. A total of 48 patients were initially selected for the study, and 8 were excluded by the non-inclusion criteria, which are described in 4.2 (Non-inclusion criteria), totaling 40 eligible patients. All patients received and signed the Informed Consent Form (TCLE) and the study was registered in the Brazilian Registry of Clinical Trials (ReBEC, Protocol No. 13872).

**Figure 2:**
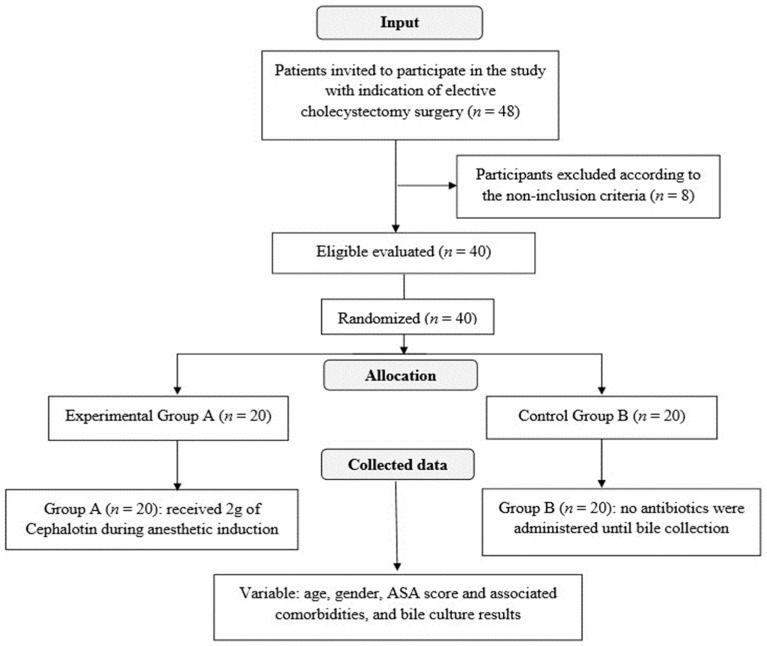
Entry flowchart and randomization of sample elements.

The following data were collected for each patient: age, gender, ASA score and associated comorbidities, and bile culture results. The input flowchart and randomization of the sample elements are shown in Figure 2.

### 4.1. Inclusion Criteria

This study included low-risk patients aged between 18 and 65 years old, diagnosed with cholelithiasis, symptomatic or not, submitted to elective laparoscopic cholecystectomy, and who agreed (in writing) to participate in the study by signing the Informed Consent Form (ASA ≤ 2).

### 4.2. Non-Inclusion Criteria

Patients with an intraoperative diagnosis of acute cholecystitis or gallbladder empyema, recent episodes of biliary tract obstruction, previous biliary pancreatitis, emergency surgery or laparotomy, patients undergoing previous procedures in the biliary tract, patients with accidental gallbladder perforation with intraoperative bile leakage, and high-risk patients were not included in the study (ASA > 2).

### 4.3. Experimental Design and Operative Procedures

The selected patients were randomly divided into 2 groups: Group A (*n* = 20), which received 2 g of Cephalotin (1st generation cephalosporin, Keflin^®^, ABL antibiotics do Brasil Ltd., Cosmópolis, Brazil) [45,46] during anesthetic induction, and Group B (*n* = 20) where no antibiotics were administered until bile collection.

The patients underwent elective laparoscopic cholecystectomy under general anesthesia in the following sequence: Propofol 10 mg/mL (Cristália—Produtos Químicos Farmacêuticos Ltd., Itapira, Brazil) intravenously with a dose of 1.5 to 2.5 mg/kg; Cisatracurium besylate 2 mg/mL (CIS^®^, Cristália Produtos Químicos Farmacêuticos, Itapira, Brazil) dose of 0.15 to 0.20 mg/kg; Fentanyl citrate 0.0785 mg/mL (Fentanest^®^, Cristália Produtos Quimicos Farmacêuticos, Itapira, Brazil) dose of 3 to 5 mcg/kg; and Remifentanil hydrochloride 2 mg (Remifas^®^, Cristália—Produtos Químicos Farmacêuticos, Itapira, Brazil) dose of 0.10 to 0.40 mcg/kg.

The surgery followed the usual steps of videolaparoscopy surgeries in gallbladder removal (available in Video S1, Supplementary Material). After general anesthesia and orotracheal intubation, the patient was placed in horizontal dorsal decubitus, antisepsis was performed with 2% alcoholic chlorhexidine (Riohex^®^, Rioquímica, São José do Rio Preto, Brazil) and sterile drapes were placed. Then, the skin was incised and pneumoperitoneum was performed by puncture of the left subcostal area with a Veress needle (Vicare^®^, São Leopoldo, Brazil), using the Laparoscopic Insufflator Fluxun 45L^®^ intelligent heater (ANVISA register No. 80370480026, Astustec medical technology, São Paulo, Brazil).

Four trocars were placed, 1 of 11 mm in the epigastric region, 1 of 11 mm in the umbilicus and 2 trocars of 5 mm in the right flank, assisted by the use of the Visun 3CMOS^®^ video camera Intelitive Endoscopic Microcamera device (Brazilian Health Regulatory Agency, ANVISA register No. 80370480027, Astustec medical technology, São Paulo, Brazil). With the electric scalpel, we dissected the cystic duct and cystic artery, which allowed the placement of metal clips in the cystic duct (2 distal and 1 proximal), with subsequent sections with surgical scissors. Metallic clips were also placed in the cystic artery (2 distal and 1 proximal) and subsequently sectioned with surgical scissors.

With the artery and cystic duct ligated, we dissected the gallbladder, separating it from the liver wall with an electric scalpel. After achieving hemostasis, the gallbladder was removed through an epigastric trocar. During the described surgical steps, ASTUS^®^ laparoscopic forceps (Astustec medical technology, São Paulo, Brazil) were used. Finally, the skin was sutured with 4-0 nylon thread (Ethicon^®^, Johnson and Johnson Company, São Paulo, Brazil) and a dressing was made.

**Figure 3:**
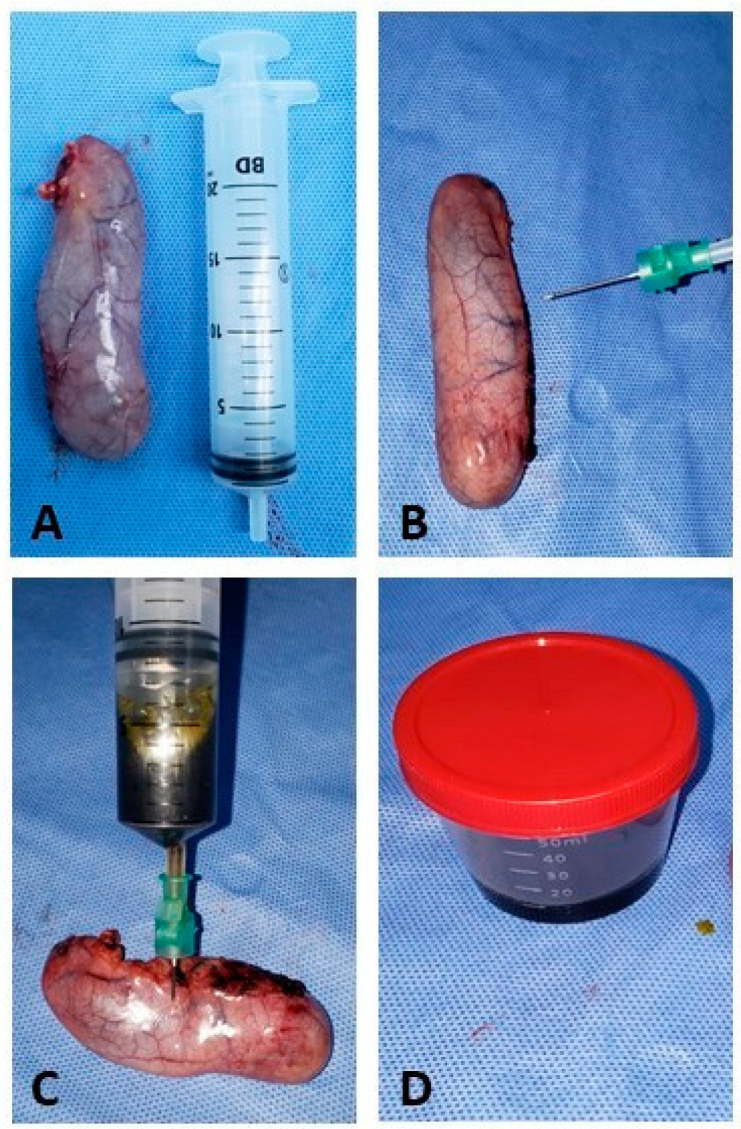
Transoperative cholecystectomy surgery. (**A**) Gallbladder removed with integrity, without ruptures or extravasation of bile content; (**B**) syringe positioning with sterile needles for bile collection; (**C**) puncture and aspiration of bile; (**D**) bile collected in a sterile bottle to be sent to the analysis laboratory.

The bile collection procedure was performed in a sterile bottle right after the removal of the gallbladder from the abdominal cavity, as shown in Figure 3.

**Figure 4:**
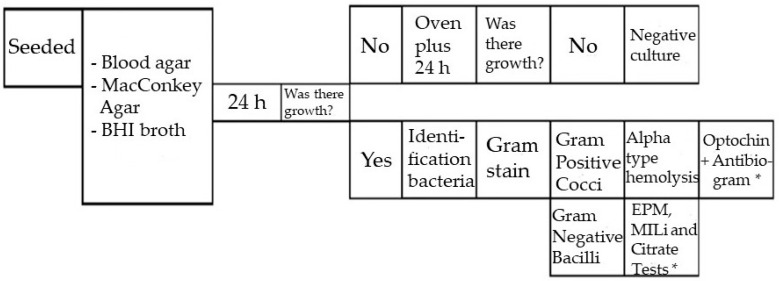
Standard operating procedure of the clinical analysis laboratory. (*) Mueller Hinton Agar, also called antibiogram, testifies whether or not a bacterium is sensitive to certain antibiotics. Standard technique recommended by the World Health Organization (WHO) for this purpose.

The flask containing the bile was sent to the Laboratory (Laboratório São Francisco, Marília, Brazil), where the bile was seeded on Blood Agar, MacConkey Agar and BHI Broth (Brain Heart Infusion) and incubated for 48 h, according to the standard operating procedure (Figure 4). 

**Figure 5:**
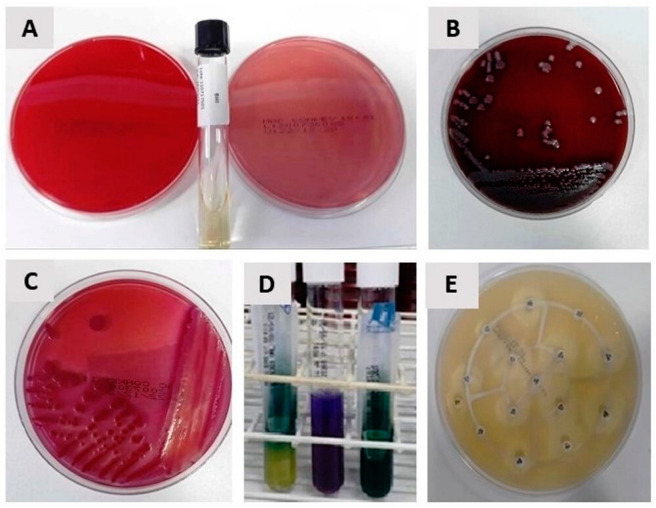
(**A**) Blood Agar, MacConkey Agar and BHI Broth; (**B**) growth on Gram-positive Cocci Blood Agar; (**C**) growth of Gram-negative bacilli on MacConkey Agar; (**D**) identification in EPM MILI and citrate; (**E**) Mueller Hinton identification (antibiogram).

After this period, the positive cultures were stained using the Gram method and an antibiogram (Mueller Hinton) was performed, as shown in Figure 5.

### 4.4. Clinical Outcome 

The patients were clinically followed up postoperatively (15 days first return and 45 days second return), and the data was accessed and evaluated in May 2023 to describe the clinical outcome. For this, they were analyzed regarding complications or postoperative complications and, if they occurred, whether they were mild, moderate or severe.

The following scores were assigned: score 1 (No)—indicates patient without complaints in the scheduled returns; score 2 (Mild)—patients complaining of pain in postoperative wounds or self-limited diarrhea; score 3 (Moderate)—need for investigation and treatment of some pathology, without antibiotic prescription; score 4 (Severe)—need to prescribe an antibiotic to treat the complication.

### 4.5. Statistical Analysis

Qualitative variables were described by relative (%) and absolute (f) frequency distribution. Quantitative variables were described by mean and standard deviation (SD). To analyze the association between the prophylactic use of the antibiotic and the positivity of bacteriobilia, the Chi-square test was performed. The Odds Ratio was calculated and its significance was determined when the 95% confidence interval (95% CI) did not include the value 1.

A Logistic Regression model was built to analyze the probability of positive culture by the Enter method. The *X*^2^ statistic was used to determine whether the variables inserted in the Logistic Regression model were significant to predict the outcome, and Nagelkerke’s R^2^ was used to determine the percentage of variation in the outcome variable explained by the model. SPSS software version 24.0 for Windows was used for all analyses, with a significance level of 5%.

## 5. Conclusions

Our clinical study aimed to compare the existence or not of bacteriobiliary in low anesthetic risk patients undergoing videolaparoscopic cholecystectomy and its correlation with the use of prophylactic antibiotics. We can conclude that patients with higher anesthetic risk (ASA II) are more likely to have bacteriobilia and may benefit from antibiotic prophylaxis when compared to patients with lower anesthetic risk (ASA I). The findings allow clinicians to reflect on the indication or not of prior antibiotic therapy, allowing a possible decrease in the use of antibiotics, consequently reducing costs and bacterial resistance to antibiotics.

## Figures and Tables

**Table 1:** Absolute (*n*) and relative (%) frequency distribution of sample characteristics by group.

Variables	Categories		Group		Total	*p* Value
			Experimental A	Control B		
Gender	Male	*n* (%)	3 (15.0)	3 (15.0)	6 (15.0)	0.669
	Female	*n* (%)	17 (85.0)	17 (85.0)	34 (85.0)	
ASA	II	*n* (%)	9 (45.0)	13 (65.0)	22 (55.0)	0.341
	I	*n* (%)	11 (55.0)	7 (35.0)	18 (45.0)	
Culture	Positive	*n* (%)	2 (10.0)	9 (45.0)	11 (27.5)	0.031 *
	Negative	*n* (%)	18 (90.0)	11 (55.0)	29 (72.5)	
Hypertension	Yes	*n* (%)	6 (30.0)	5 (25.0)	11 (27.5)	0.500
	No	*n* (%)	14 (70.0)	15 (75.0)	29 (72.5)	
Diabetes	Yes	*n* (%)	3 (15.0)	4 (20.0)	7 (17.5)	0.500
	No	*n* (%)	17 (85.0)	16 (80.0)	33 (82.5)	
Hypothyroidism	Yes	*n* (%)	3 (15.0)	6 (30.0)	9 (22.5)	0.451
	No	*n* (%)	17 (85.0)	14 (70.0)	31 (77.5)	
Smoking	Yes	*n* (%)	4 (20.0)	4 (20.0)	8 (20.0)	0.653
	No	*n* (%)	16 (80.0)	16 (80.0)	32 (80.0)	
Obesity	Yes	*n* (%)	2 (10.0)	0 (0.0)	2 (5.0)	0.487
	No	*n* (%)	18 (90.0)	20 (100.0)	38 (95.0)	
Complications	Yes	*n* (%)	11 (55.0)	11 (55.0)	22 (55.0)	0.999
	No	*n* (%)	9 (45.0)	9 (45.0)	18 (45.0)	

Note: * indicates a significant difference in the distribution of the response categories by the Chi-square test for proportion to *p* value ≤ 0.05.

**Table 2:** Analysis of the association of absolute (*n*) and relative (%) frequency distribution of positive culture with antibiotic use, gender, ASA and morbidities.

			Culture		*X* ^2^	Odds	CI 95% (Odds)	
			Positive	Negative	*p* Value		Inferior	Superior
ASA	II	*n*	10	12	0.006 *	14.1 †	1.59	125.8
		%	90.9%	41.4%				
	I	*n*	1	17				
		%	9.1%	58.6%				
Antibiotic	Yes	*n*	2	18	0.014 *	0.13 †	0.02	0.74
		%	18.2%	62.1%				
	No	*n*	9	11				
		%	81.8%	37.9%				
Gender	Male	*n*	1	5	0.524	0.48	0.05	4.64
		%	9.1%	17.2%				
	Female	*n*	10	24				
		%	90.9%	82.8%				
Hypertension	Yes	*n*	4	7	0.445	0.17	0.40	8.00
		%	36.4%	24.1%				
	No	*n*	7	22				
		%	63.6%	75.9%				
Diabetes	Yes	*n*	2	5	0.945	1.06	0.17	6.51
		%	18.2%	17.2%				
	No	*n*	9	24				
		%	81.8%	82.8%				
Hypothyroidism	Yes	*n*	4	5	0.202	2.74	0.57	13.00
		%	36.4%	17.2%				
	No	*n*	7	24				
		%	63.6%	82.8%				
Smoking	Yes	*n*	4	4	0.116	3.57	0.70	18.00
		%	36.4%	13.8%				
	No	*n*	7	25				
		%	63.6%	86.2%				
Obesity	Yes	*n*	0	2	0.378	-	-	-
		%	0.0%	6.9%				
	No	*n*	11	27				
		%	100.0%	93.1%				
Complications	Yes	*n*	13	9	0.570	1.44	0.41	5.06
		%	59.1%	50%				
	No	*n*	9	9				
		%	40.9%	50%				

Note: * indicates significant association with positive culture by Chi-square test (*X*^2^) for *p* value ≤ 0.05. † indicates significant value for Odds based on 95% confidence interval (95% CI).

**Table 3:** Logistic regression analysis to analyze the effect of ASA and antibiotics on the probability of having a positive culture.

Variables		B	Odds	IC 95% Odds		*p* Value	Model	
Dependent	Independent			Inferior	Superior		*p* Value	R^2^
Culture (positive)	ASA	2.59	13.30	1.38	128.33	0.025 *	0.001 †	0.426
	Antibiotic	−1.92	0.15	0.02	0.92	0.041 *		
	Constant	0.72	2.06			0.723		

Note: B regression coefficient; Odds Ratio (Odds); 95% CI 95% confidence interval for Odds. * *p* value ≤ 0.05 significant effect of the variable by Wald statistics; † *p* value ≤ 0.05 indicates that the model variables are significant to predict the dependent variable by the Chi-square test; ASA (at risk); antibiotic (gift). Accuracy of Nagelkerke’s R^2^.

**Table 4:** Analysis of the association of absolute (*n*) and relative (%) frequency distribution of the rate of complications with positive culture, use of antibiotics and ASA.

Variables	Categories		Complication Rate				*X* ^2^
			No (Score 1)	Mild (Score 2)	Moderate (Score 3)	Severe (Score 4)	*p* Value
ASA	II	*n* (%)	13 (59.1)	3 (33.3)	2 (50.0)	4 (80.0)	0.769
	I	*n* (%)	9 (40.9)	6 (66.7)	2 (50.0)	1 (20.0)	
Group	Experimental A	*n* (%)	11 (50.0)	6 (66.7)	2 (50.0)	1 (20.0)	0.374
	Control B	*n* (%)	11 (50.0)	3 (33.3)	2 (50.0)	4 (80.0)	
Culture	Positive	*n* (%)	7 (31.8)	1 (11.1)	1 (25.0)	2 (40.0)	0.947
	Negative	*n* (%)	15 (68.2)	8 (88.9)	3 (75.0)	3 (60.0)	

Note: *p* value calculated by the Chi-square test. Score 1—indicates patient without complaints in the scheduled returns; score 2—patients complaining of pain in postoperative wounds or self-limited diarrhea; score 3—need for investigation and treatment of some pathology, without antibiotic prescription; score 4—need to prescribe an antibiotic to treat the complication.

**Table 5:** Calculation of effect size and study power based on results (Figure S1).

Group (Antibiotic)		Culture		Total	*X* ^2^	Effect Size	Power
		Positive	Negative		*p* Value		
Experimental A	*n* (%)	2 (5.0)	18 (45.0)	20 (50.0)	0.014	0.57	89.00%
Control B	*n* (%)	9 (22.5)	11 (27.5)	20 (50.0)			
Total	*n* (%)	11 (27.5)	29 (72.5)	40 (100.0)			

## Data Availability

Data are contained within the article.

## Supplementary Materials

The following supporting information can be downloaded at: https://www.mdpi.com/article/10.3390/antibiotics12101480/s1. Figure S1: calculation of effect size and study power based on results. Video S1: laparoscopic cholecystectomy.
