# Supplementary material for: Incidence of Bacteriobilia and the Correlation with Antibioticoprophylaxis in Low-Risk Patients Submitted to Elective Videolaparoscopic Cholecystectomy: A Randomized Clinical Trial
Source: Antibiotics (Basel). 2023 Sep 25;12(10):1480. doi: 10.3390/antibiotics12101480 (PMC10604456; doi:10.3390/antibiotics12101480)
Supplement: Supplementary file 1 [file antibiotics-12-01480-s001.zip › antibiotics-2538946-supplementary.pdf]

Figure S1: Calculation of effect size and study power based on results

Group * Culture Crosstabulation

			Culture		
			Positive	Negative	Total
Group	Experimental A	Count	2	18	20
		% of Total	5.0%	45.0%	50.0%
	Control B	Count	9	11	20
		% of Total	22.5%	27.5%	50.0%
Total		Count	11	29	40
		% of Total	27.5%	72.5%	100.0%

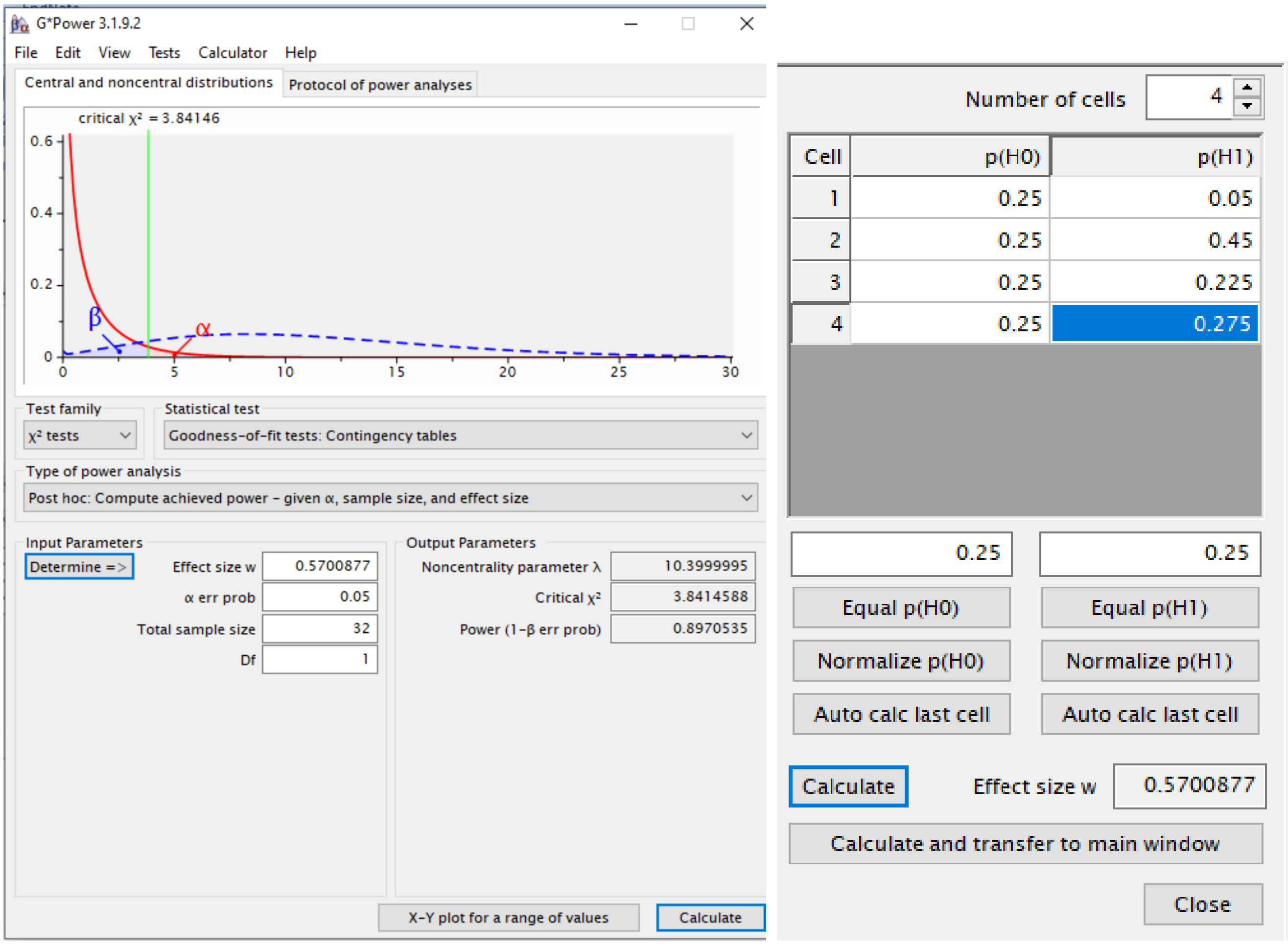

Initial calculation

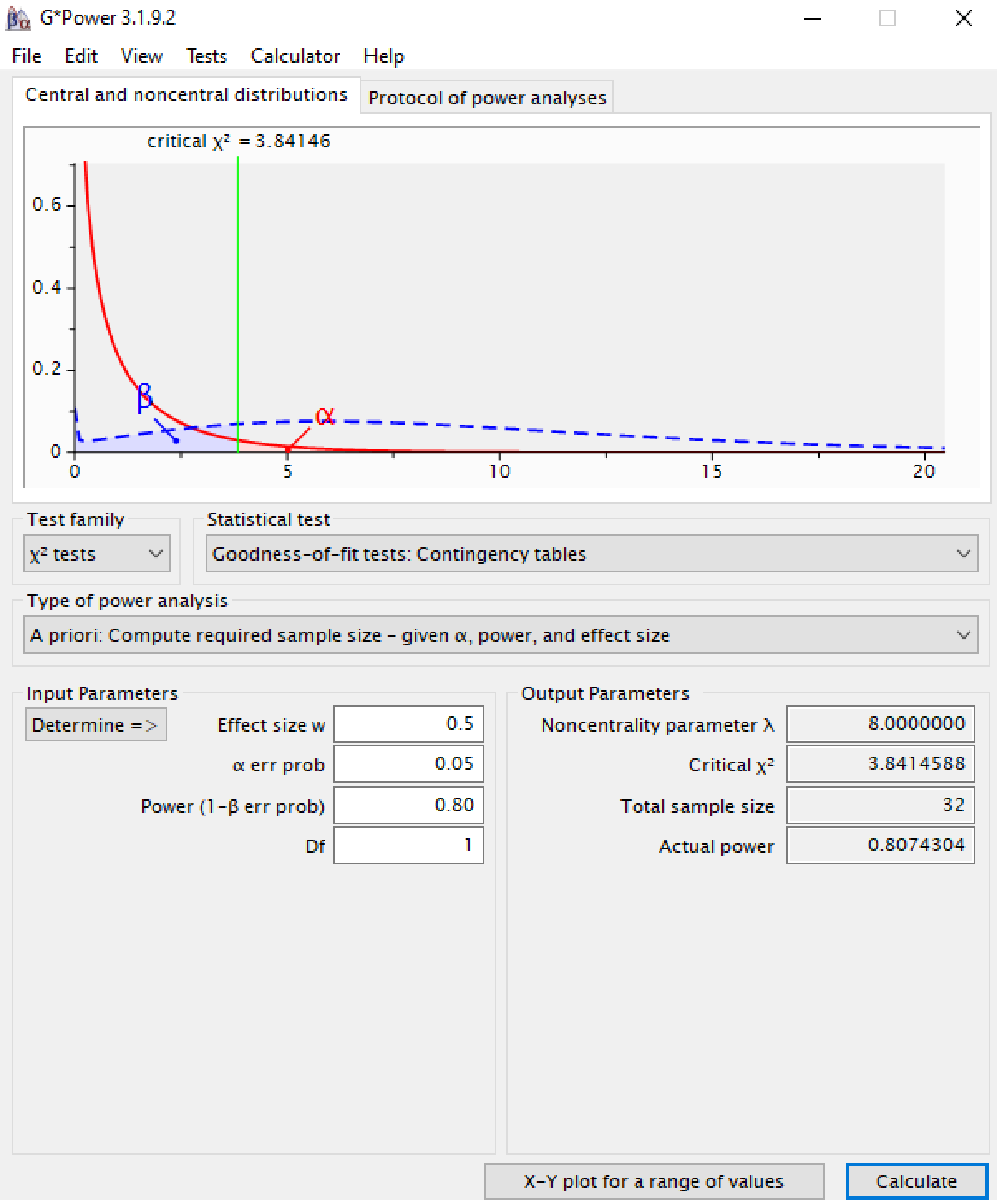
G*Power 3.1.9.2
File Edit View Tests Calculator Help
Central and noncentral distributions
Protocol of power analyses
critical χ² = 3.84146
0.6
0.4
0.2
0
0
5
10
15
20
β
α
Test family
χ² tests
Statistical test
Goodness-of-fit tests: Contingency tables
Type of power analysis
A priori: Compute required sample size – given α, power, and effect size
Input Parameters
Determine =>
Effect size w
0.5
α err prob
0.05
Power (1-β err prob)
0.80
Df
1
Output Parameters
Noncentrality parameter λ
8.0000000
Critical χ²
3.8414588
Total sample size
32
Actual power
0.8074304
X-Y plot for a range of values
Calculate
